# A glycolytic phenotype is associated with prostate cancer progression and aggressiveness: a role for monocarboxylate transporters as metabolic targets for therapy

**DOI:** 10.1002/path.4547

**Published:** 2015-07-20

**Authors:** Nelma Pertega‐Gomes, Sergio Felisbino, Charlie E Massie, Jose R Vizcaino, Ricardo Coelho, Chiranjeevi Sandi, Susana Simoes‐Sousa, Sarah Jurmeister, Antonio Ramos‐Montoya, Mohammad Asim, Maxine Tran, Elsa Oliveira, Alexandre Lobo da Cunha, Valdemar Maximo, Fatima Baltazar, David E Neal, Lee GD Fryer

**Affiliations:** ^1^ Uro‐oncology Research GroupCancer Research UK (CRUK) Cambridge InstituteCambridgeUK; ^2^ Department of MorphologyInstitute of Biosciences, Sao Paulo State University (UNESP)BotucatuBrazil; ^3^ Department of PathologyCentro Hospitalar do PortoPortugal; ^4^ Institute of Molecular Pathology and Immunology of the University of Porto (IPATIMUP)Portugal; ^5^ Life and Health Sciences Research Institute (ICVS)School of Health Sciences,University of MinhoBragaPortugal; ^6^ ICVS/3Bs–PT Government Associate LaboratoryBraga/GuimaraesPortugal; ^7^ Laboratory of Cell Biology, Institute of Biomedical Sciences Abel Salazar (ICBAS)University of PortoPortugal; ^8^ Department of Pathology and OncologyMedical Faculty of the University of PortoPortugal; ^9^ Department of UrologyUniversity of Cambridge, and S4, Department of Oncology, Addenbrooke's HospitalCambridgeUK

**Keywords:** prostate cancer, cell metabolism, monocarboxylate transporters, poor prognosis markers, metabolic targets

## Abstract

Metabolic adaptation is considered an emerging hallmark of cancer, whereby cancer cells exhibit high rates of glucose consumption with consequent lactate production. To ensure rapid efflux of lactate, most cancer cells express high levels of monocarboxylate transporters (MCTs), which therefore may constitute suitable therapeutic targets. The impact of MCT inhibition, along with the clinical impact of altered cellular metabolism during prostate cancer (PCa) initiation and progression, has not been described. Using a large cohort of human prostate tissues of different grades, in silico data, in vitro and ex vivo studies, we demonstrate the metabolic heterogeneity of PCa and its clinical relevance. We show an increased glycolytic phenotype in advanced stages of PCa and its correlation with poor prognosis. Finally, we present evidence supporting MCTs as suitable targets in PCa, affecting not only cancer cell proliferation and survival but also the expression of a number of hypoxia‐inducible factor target genes associated with poor prognosis. Herein, we suggest that patients with highly glycolytic tumours have poorer outcome, supporting the notion of targeting glycolytic tumour cells in prostate cancer through the use of MCT inhibitors. © 2015 Authors. Journal of Pathology published by John Wiley & Sons Ltd on behalf of Pathological Society of Great Britain and Ireland.

## Introduction

Changes in cellular metabolism have recently emerged as a hallmark of cancer 1, 2 leading to an increase in the number of novel therapies targeting cancer metabolism 3, 4, 5. Fluorodeoxyglucose positron emission tomography (FDG‐PET) for malignancy has been introduced as a technique for metabolic imaging, based on the fact that cancer tissue consumes a large amount of glucose as an energy source and fluorodeoxyglucose [^I8^F] enters cancer cells through the same transport route as glucose. However, there appears to be a consensus that FDG‐PET is not particularly useful for the detection or metabolic grading of PCa, suggesting that in PCa, contrary to other malignant neoplasms, glucose consumption is not directly associated with malignancy 6. However, some studies have reported that the accumulation of FDG in the prostate is higher in advanced stages of PCa, rather than in early stages 7. Although much of metabolic cancer research focuses on the role of glycolysis, it has recently become apparent that the tricarboxylic acid (TCA) cycle and oxidative phosphorylation (OXPHOS) also have major roles in many cancer types, including prostate 8, 9, 10, 11, 12. These data indicate that PCas may exhibit unique metabolic profiles; however, the clinical impact of these metabolic profiles is not known.

Previous work has suggested a role for monocarboxylate transporters (MCTs) at the diagnostic and prognostic level in PCa 13, 14, 15, 16. MCTs are transmembrane proteins involved in the transport of important monocarboxylates, such as lactate, and it has been reported that most cancer cells express high levels of MCTs to ensure rapid efflux of the lactate produced 17. They facilitate the efflux of lactate while they also contribute to the preservation of intracellular pH, by co‐transporting a proton 18. MCTs have been recently explored as targets for cancer therapy 19, 20, 21, 22. Kim *et al* 23 reported an increased necrotic fraction with no variations in prostate tumour volume following MCTs inhibition; however, these results were obtained using α‐cyano‐4‐hydroxycinnamate (CHC), which is a non‐specific inhibitor of lactate transport.

Here, we detail the expression of MCTs as well as the expression of a wide range of glycolysis‐related proteins in a large cohort of human prostate samples, including metastatic PCa and across different *in vitro* models of PCa progression, and demonstrate the impact of MCTs inhibition in PCa cells *in vitro* and *ex vivo*.

## Materials and methods

### Patient samples and tissue microarray construction

Samples, including 203 non‐neoplastic, 176 high‐grade prostatic intraepithelial neoplasia (PIN) and 480 neoplastic tissues were organized into tissue microarray blocks (TMAs). Benign samples from 12 patients undergoing radical cystoprostatectomy and 10 metastatic PCa cases were obtained from clinical biopsy samples. The present study was previously approved by the local ethical review committee.

### Immunohistochemistry (IHC) staining and analysis

IHC technique was performed according to the information given in Table 1, as previously described 9. For each treatment group (vehicle or drug), a total number of 30–50 randomly chosen images were used for the analysis.

**Table 1:** Details of the immunohistochemical procedure used to analyse the expression of different proteins in human prostate samples

Protein	Antibody	Company	Antibody dilution	Positive control	Incubation period	Detection system
MCT4	sc‐50329	Santa Cruz Biotechnology	1:500	Colon tumour	Overnight	R.T.U. Vectastain Universal Elite ABC Kit, Vector, EUA
GLUT1	ab 15309	Abcam	1:2000	Head and neck tumour		
CAIX	ab 15086	Abcam	1:2000	Stomach		
MCT1	sc‐365501	Santa Cruz Biotechnology	1:500	Colon tumour	Overnight	R.T.U. Vectastain Universal Elite ABC Kit, Vector, EUA
HIF‐1α	610958	BD Biosciences	1:100	Glioblastoma		
LDH5	ab 53010	Abcam	1:1000	Colon tumour	2 h	
HK2	ab104836	Abcam	1:750	Colon tumour		
PDK1	ab110025	Abcam	1:500	Stomach		
ki67	M7249	Dako	1:50			
CC3	9664	Cell Signaling	1:2000			

### Western blot analysis

Western blot analysis was performed for protein expression using antibodies (Santa Cruz Biotechnology, CA, USA) for MCT1 (sc‐365501, 1:500 dilution), MCT4 (sc‐50329, 1:500 dilution), Actin (sc‐1616, 1:5000 dilution), TOM20 (sc‐11415, 1:5000 dilution) and UCP‐2 (sc‐6525, 1:400 dilution).

### Cell culture and drugs

Prostate cell lines were obtained from the American Type Culture Collection (ATCC, Manassas, VT, USA). R1881 (Sigma, Gillingham, Dorset, UK) was used at a concentration of 1 nm. For hypoxia treatment, cell cultures were incubated in 1% O_2_. For experiments using lactate, cells were exposed to 10 mm sodium l‐lactate, a concentration that corresponds to the level of lactate most commonly detected in human tumours 11.

### siRNA transfection

siRNAs were purchased from Qiagen (Manchester, UK) (Table 2) and transfected using Lipofectamine RNAiMAX (Invitrogen, Life Technologies, Paisley, North Renfrewshire, UK). Final siRNA concentrations of 10 nm were used.

**Table 2:** List of siRNAs used for gene silencing

Target	siRNA	Catalogue no.
SLC16A1 (MCT1)	Hs_SLC16A1_6 siRNA	SI032466362
	Hs_SLC16A1_7 siRNA	SI03246404
SLC16A3 (MCT4)	Hs_SLC16A3_4 siRNA	SI00720440
	Hs_SLC16A3_5 siRNA	SI04134774

All siRNAs were purchased from Qiagen.

### Metabolic profiling (extracellular glucose and lactate measurements)

Cells were plated in 24‐well plates until confluent. Glucose consumption and lactate production were quantified before and after treatment using commercial kits, as previously described 12.

### ECAR and OCR measurements

ECAR reflective of the rate of glycolysis, and OCR, reflective of the rate of OXPHOS, were measured using a Seahorse Bioscience (Copenhagen, Denmark) XF24 analyser. Cells were plated in Seahorse XF24 plates and incubated for 48 h. For knockdown experiments, cells were transfected with the indicated siRNAs overnight before treatment.

### Analysis of cell respiratory efficiency

The activity of the mitochondrial complex I (NADH:ubiquinone oxidoreductase) was evaluated enzymatically using the Complex I Enzyme Activity Microplate Assay Kit (ab109721, Abcam, Cambridge, UK), according to the manufacturer's instructions. The global cellular ATP levels were quantified using a Luminescent ATP Detection Assay Kit (ab113849, Abcam).

### Cell proliferation and viability assay

Reverse transfection was carried out on the day of seeding. Cell number and viability were determined using a Beckman Coulter™ (High Wycombe, Bucks, UK) Vi‐Cell.

### Cell‐cycle analysis

Adherent cells were fixed with ice‐cold 70% ethanol and incubated with 50 µg/ml propidium iodide and 10 µg/ml RNaseA in phosphate‐buffered saline (PBS) for 30 min at 37° in the dark. Propidium iodide staining intensity was detected by flow cytometry (BDFacsCalibur, Oxford, UK).

### Quantitative real‐time PCR (qRT–PCR)

For RNA isolation, the RNeasy Plus Mini Kit (Qiagen) was used according to the manufacturer's instructions. cDNA was synthesized using a High Capacity cDNA Reverse Transcripton Kit (Applied Biosystems, Life Technologies, Paisley, North Renfrewshire, UK). qRT–PCR reactions were performed in triplicate in 384‐well plates in cell lines. For each reaction, 1 ng cDNA and 5 µl Fast SYBR Green Master Mix (Applied Biosystems) were used in a 10 µl reaction volume. mRNA expression was normalized to retention in endoplasmic reticulum 1 (RER1), using the 2^−*ΔΔC*t^ method.

### Ultrastructural studies

Samples were fixed in 2.5% glutaraldhyde in 0.1 m sodium cacodylate buffer, washed in the same buffer, post‐fixed in 1% OsO_4_ in buffer, dehydrated in ethanol and embedded in Epon. Ultrathin sections were collected on 300 mesh copper grids and stained with 3% aqueous uranyl acetate and lead citrate before being observed in a transmission electron microscope (JEOL 100 CX II, Atlanta, Georgia, UK) operated at 60 kV.

### Statistical analysis

Data from human tissue samples were analysed with SPSS statistical software v. 18.0 (SPSS, IBM, London, UK) using Pearson's χ^2^ test, with the threshold for significance being *p* ≤ 0.05. For *in vitro* studies, GraphPad Prism 5 software was used with Student's *t*‐test, considering significant values to be *p* ≤ 0.05.

### Ex vivo cultures

Samples from 10 wild‐type animals, eight p53 transgenic mice with PIN lesions, five p53 transgenic mice with undifferentiated adenocarcinoma, nine *PTEN*‐knockout mice with high‐grade prostatic intra‐epithelial neoplasia (PIN), 12 *PTEN*‐knockout mice with micro‐invasive adenocarcinoma and 14 *PTEN*‐knockout mice with moderated and undifferentiated adenocarcinoma for the four prostate lobes was used. All experiments were conducted according to the UK Home Office Regulations and protocols approved under Project License No. 80‐2435. Tumours were collected and grown *ex vivo*. A solution containing collagen, Matrigel and RPMI was prepared, added to nylon sheets and incubated; 10 ml 1% glutaraldehyde in PBS was added to the sheets. After 1 h of incubation at 4 °C, the nylon sheets were washed with PBS and with 10% RPMI and incubated in 10% RPMI overnight. Grids were plated in a six‐well dish and the coated nylon sheet placed on the grids. The well was filled with medium containing gentamycin and treated with 50 µm AR‐C155858. Tumours were harvested 5 days later.

## Results

### The expression of glycolysis‐related proteins correlates with prostate cancer progression in patients

**Figure 1:**
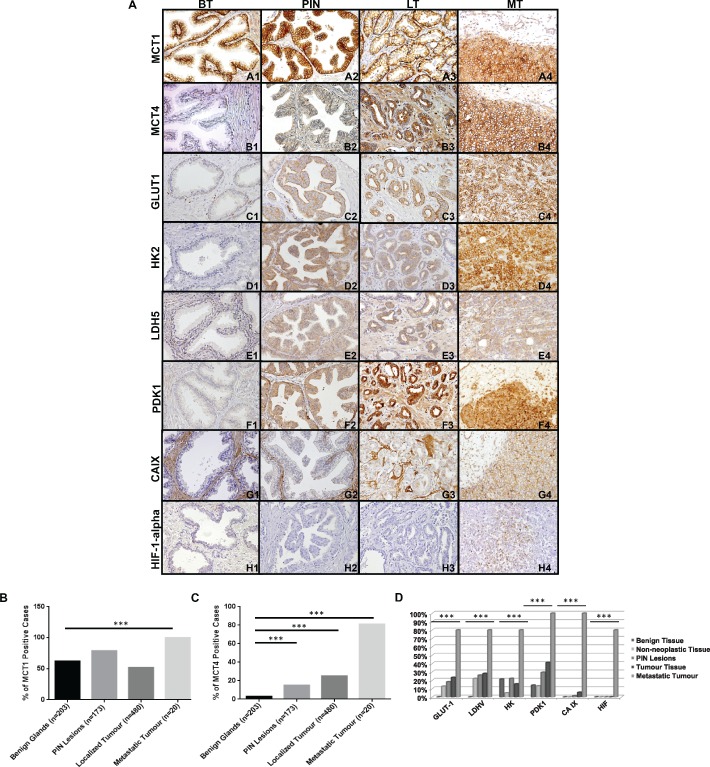
MCTs and glycolysis‐related protein expression is increased in PIN lesions, PCa and metastatic tissue. (A) Immunohistochemical expression of MCT1, MCT4, GLUT1, HK2, LDH5, PDK1, CAIX and HIF‐1α in benign tissue (BT), PIN lesions (PIN), tissue from localized prostate tumour (LT) and metastatic tumour tissue (MT); magnification = ×200). (B–D) Representation of positive cases for MCT1, MCT4 and glycolysis‐related proteins in benign tissue, non‐neoplastic tissue, PIN lesions, localized tumours and metastatic tumours (***p < 0.001)

Figure 1A shows the immunohistochemical expression of MCT1, MCT4, glucose transporter 1 (GLUT1), hexokinase 2 (HK2), lactate dehydrogenase 5 (LDH5), pyruvate dehydrogenase kinase 1 (PDK1), carbonic anhydrase IX (CAIX) and hypoxia‐inducible factor 1α (HIF‐1α) in benign prostate tissue (BT), prostatic intra‐epithelial neoplasia (PIN), localized prostate tumour tissue (LT) and metastatic tumour tissue (MT). In general, we observed that normal prostate epithelium was either negative or weakly positive for MCT4 and glycolysis‐related proteins, with the exception of MCT1, whereas PIN lesions and adenocarcinomas showed intense staining in the majority of cases. The most striking findings were obtained for MCT4 (Figure 1A, B1–B4) and CAIX (Figure 1A, G1–G4), where an increase in the intensity and extension of the staining from LT to MT was evident. Also, HIF‐1α only appeared in the nucleus of the cells in metastatic samples (Figure 1A, H1–H4). Quantification of the overall fraction of positive cases for the expression of MCT1, MCT4 and glycolysis‐related proteins are shown in Figure 1B–D. In contrast to MCT1 (Figure 1B) we observed that the overall percentage of positive cases for the expression of MCT4 (Figure 1C) and glycolysis‐related proteins (Figure1D) increase from non‐neoplastic tissue to PIN lesions and localized tumour, being most highly expressed in almost all metastatic PCa tissues analysed.

### The expression of MCT4 and proteins involved in glycolytic metabolism is associated with poor prognosis

**Figure 2:**
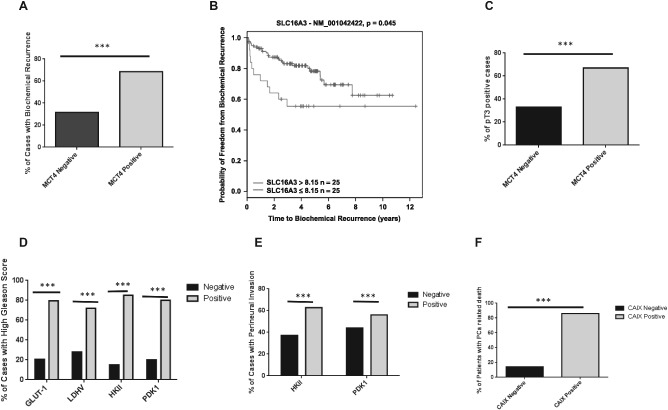
The expression of MCT4 and glycolysis‐related proteins is associated with poor prognosis in PCa. (A) MCT4 expression at protein level in human prostate cancer samples is associated with the presence of biochemical recurrence after surgery. (B) Kaplan–Meyer plots for SLC16A3 (MCT4), based on Glinsky et al 24; recursive partitioning was performed using a galaxy‐based CRI Bioinformatics Core Facility tool. (C) Pathological stage of the tumour (pT3); the expression of glycolysis‐related proteins was associated with (D) cases of higher Gleason score, (E) presence of perineural invasion and (F) prostate cancer‐related death (***p < 0.001)

**Figure 3:**
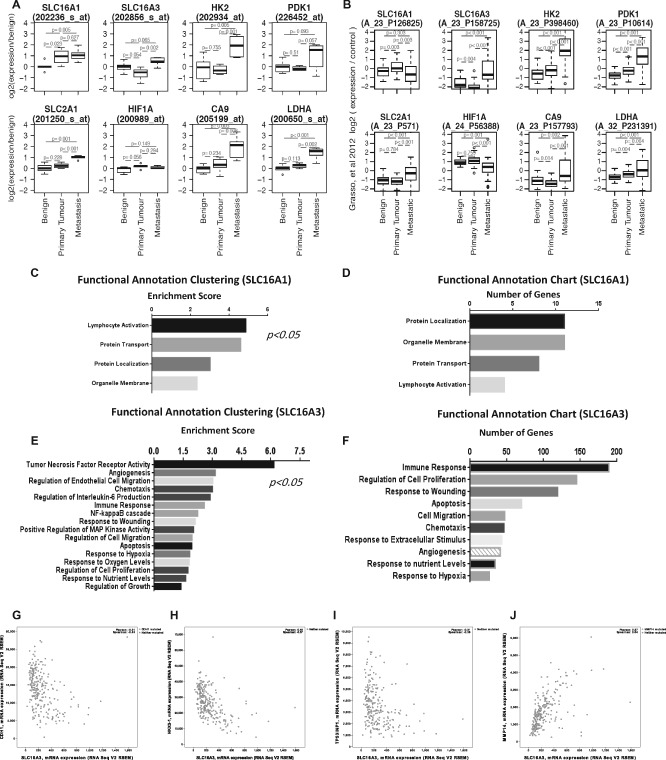
Analysis of microarray expression data for SLC16A1, SLC16A3 and glycolysis‐related genes from the Oncomine database. Expression is presented for two different datasets, (A) Varambally et al and (B) Grasso et al, representing different detection probes. (C–F) Expression profiles of the co‐expressed genes SLC16A1 and SLC16A3 were obtained from the Cancer Genome Atlas (TCGA) and clustered by functional role and signalling pathways, using the DAVID in silico tool: the left panels (C, E) represent the functional clusters organized by enrichment score, and the right panels (D, F) represent the signalling pathway analysis. SLC16A3 co‐expressed genes are associated with pathways involved in tumour aggressiveness, viz in immune cell response, positive cell cycle regulation, cell motility and chemotaxis. (G–J) In silico analysis indicates an inverse correlation between SLC16A3 and the epithelial markers E‐cadherin (CDH1) (G), NKX3.1 (H), TP53 (I) and a direct correlation with several genes involved in the epithelial–mesenchymal transition (EMT), such as MMP14 (J)

We observed that MCT4 expression was associated with poor prognosis, ie the presence of biochemical recurrence after surgery based on protein expression (Figure 2A) and mRNA levels based on the Glinsky dataset (Figure 2B) 24. MCT4 protein expression was also associated with higher tumour stage (Figure 2C). The expression of glycolysis‐related proteins in localized prostate tumours was associated with important prognostic parameters, including Gleason score (Figure 2D), presence of perineural invasion (Figure 2E) and patient survival status (Figure 2F). To corroborate our findings, we extended the analysis to microarray profiling datasets of PCa tissues available on the Oncomine database 25. mRNA expression of SLC16A1 (MCT1), SLC16A3 (MCT4) and a number of glycolysis‐related proteins was analysed in two independent PCa datasets, Varambally *et al* 26 and Grasso *et al* 27 (Figure 3A, B). We found that SLC16A1 (MCT1), SLC16A3 (MCT4) and the glycolysis‐related proteins were significantly over‐expressed in primary and metastatic PCa. After this observation we clustered genes co‐expressed with SLC16A1 and SLC16A3 in prostate tissues available at the Cancer Genome Atlas (TCGA) database by their functional role and importance in signal transduction pathways, using the DAVID bioinformatics tool (Figure 3C–F). We found that the majority of genes co‐expressed with SLC16A1 were genes correlated with normal biological processes of cells, while genes co‐expressed with SLC16A3 were functionally clustered in the categories considered relevant for tumour progression and development. A sub‐analysis of signalling pathways revealed that SLC16A3‐co‐expressed genes are grouped in pathways associated with higher aggressiveness, viz immune response and regulation of cell proliferation and migration (Figure 3F). Importantly, *in silico* analysis indicates inverse correlation between SLC16A3 and the epithelial markers E‐cadherin, NKX3.1 and TP53INP1, and a direct correlation with MMP14, involved in the epithelial–mesenchymal transition (EMT) process (Figure 3G–J), suggesting a possible role in metastasis.

### Different models of prostate cancer progression recapitulate expression profiles of human PCa tissues and exhibit a distinct metabolic phenotype

**Figure 4:**
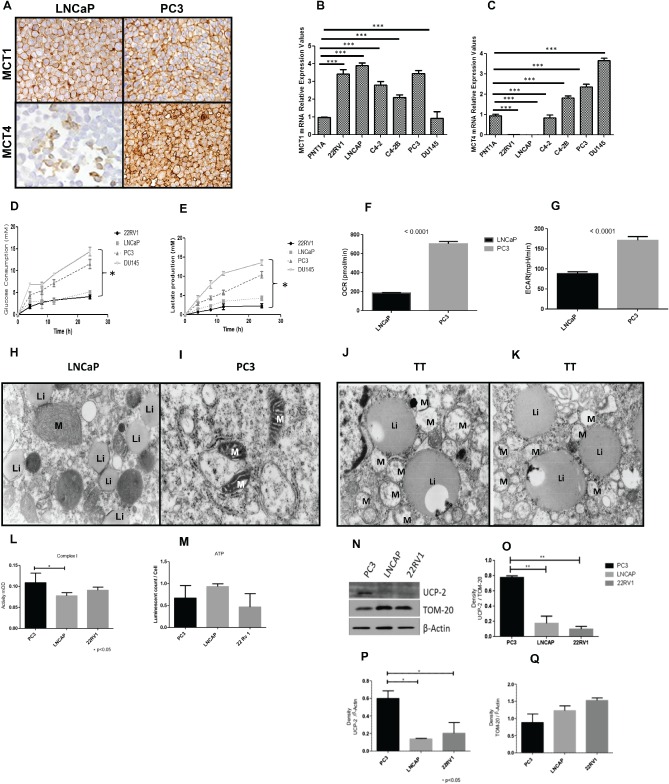
Distinct in vitro models of PCa cell lines exhibit different metabolic behaviours. (A) Immunohistochemical expression of MCT1 and MCT4 in distinct in vitro models of prostate cancer progression. (B, C) mRNA expression levels of MCT1 and MCT4 in prostate‐derived cell lines; for RNA quantification, n = 3; data expressed as ± SD (n = 3); ***p < 0.001. (D–G) Metabolic characterization of prostate cell lines, based on (D) glucose consumption and (E) lactate production; (F) oxygen consumption rates (OCRs) and (G) extracellular acidification rates (ECAR) are also represented for these different models of prostate cancer progression. (H, I) Electron micrographs of LNCaP and PC3 cell lines: (H) in LNCaP, mitochondria (M) and lipidic inclusions (Li) were observed distributed randomly in the cytoplasm of cells; (I) in PC3 cells, small, atypical mitochondria (M) were observed and no lipidic inclusions were seen; magnification = ×16 000). (J, K) Cells collected from tumour localized to the prostate showed structures similar to those of LNCaPs (magnification = ×16 000). (L–Q) Analysis of Complex I activity in LNCaP, 22RV1 and PC3 PCa cells: activity mOD (milli‐units of optical density) was measured at 450 nm; data correspond to two biological replicates performed in duplicate; *p < 0.05: (L, M) analysis of global ATP levels; data correspond to three biological experiments, each performed in triplicate; data were subjected to one‐way ANOVA and Tukey test; (N) western blot analysis for UCP‐2 protein, β‐actin as an exogenous control and TOM‐20 as a control for mitochondrial loading; a representative immunoblot of two experiments is shown; (O) quantification of UCP‐2 protein in the cell lines, expressed as density of UCP‐2 band/density of TOM‐20; (P) quantification of UCP‐2 protein in the cell lines, expressed as density of UCP‐2 band/density of β‐actin; (Q) quantification of TOM‐20 protein in the cell lines, expressed as density of TOM‐20 band/density of β‐actin; data were subjected to one‐way ANOVA and a posterior Tukey test; *p < 0.01

MCT4 expression was more evident at the plasma membrane of PC3 cells than in LNCaP cells, whereas MCT1 staining was similar in both models (Figure 4A). mRNA levels for *MCT1* and *MCT4* were readily detectable by qRT–PCR in cell lines (Figure 4B–C) and both MCT1 and MCT4 expression varied depending upon the cell line. *MCT4* mRNA levels clearly show a strong correlation with the aggressiveness of the PCa cell model (Figure 4C). Also, there was a clear difference in metabolic behaviour between the low‐ and high‐tumourigenic lines. PC3 and DU145 cells exhibited higher levels of glucose consumption (Figure 4D) and lactate production (Figure 4E) when compared to 22RV1 and LNCaP cells. Additionally, we measured the oxygen consumption rate (OCR) (Figure 4F) with simultaneous measurement of the extracellular acidification rate (ECAR) (Figure 4G). These results interestingly showed that although the highly metastatic model PC3 cells exhibited higher levels of ECAR, it also showed an increased oxygen consumption rate (OCR) compared to the lower tumourigenic LNCaP cells.

Our previous observation that, while PC3 cells exhibit higher levels of glucose consumption and lactate production as well as higher ECAR rates, they also show higher OCR levels, led us to analyse different cell lines at the ultrastructural level to look for differences at the mitochondrion level. Electron micrographs of LNCaP (Figure 4H), PC3 (Figure 4I) and two human PCa samples from localized tumour (TT) (Figure 4J, K) are represented. We observed that in both LNCaP cells and human samples (Figure 4H, J, K), the cells were packed with lipid bodies. Also, there was a difference in mitochondrial morphology, ie the mitochondria of PC3 cells were smaller and had an atrophic appearance, with sparse and poorly formed cristae.

Investigation of Complex I activity in prostate cell lines showed that PC3 cells have increased activity (Figure 4L). However, this increased Complex I activity was not reflected in global cellular ATP levels (Figure 4M). This suggests that the proton electrochemical gradient across the inner mitochondrial membrane (IMM) was being dissipated and not utilized for ATP production. In fact, when we looked at the expression levels of the mitochondrial uncoupling protein 2 (UCP2), we verified that it was significantly higher in PC3 cells, using both β‐actin (*p =* 0.02) as the total protein loading control, and the translocase of outer mitochondrial membrane 20 homologue (TOMM20; *p <* 0.01) as a mitochondrial protein loading control (Figure 4N–Q). Taken together, these observations point to inefficient respiration in the highly metastatic model, ie PC3 compared to the low metastatic models.

### SLC16A1 and SLC16A3 gene expression is increased under hypoxia but independent of androgen stimulation

**Figure 5:**
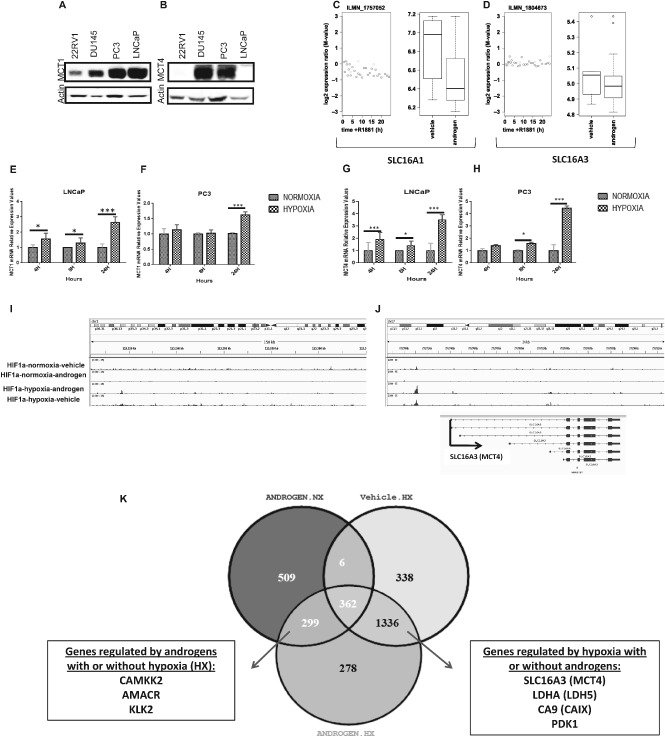
(A, B) Western blot analysis of MCT1 and MCT4, showing different levels of expression in different prostate cancer cell lines. (C–F) Effect of androgen stimulation and inhibition on MCTs expression: (C, D) plots from the expression array with androgen treatment time‐course; (E–H) effect of hypoxic conditions (1% O_2_) on MCTs expression; levels of MCT1 (E, F) and MCT4 (G, H) in PCa cells under normoxic (NX) and hypoxic (HX) conditions; aata expressed as ± SD; n = 3; *p < 0.05. (I, J) Integrated Genome Browser view of ChIP‐Seq enrichment profiles of SLC16A1 and SLC16A3 in LNCaP cell lines. (K) Venn diagram showing the overlap between genes expressed by LNCaP cell lines under different oxygen and hormone conditions

Since the androgen receptor (AR) is an important regulator of metabolic pathways in PCa, regulating the expression of various genes involved in metabolism 28, we sought to determine potential cross‐regulation between MCTs and AR signalling. We found that MCT1 levels were higher in PC3 and LNCaP cells compared to DU145 and 22RV1 cell lines (Figure 5A). MCT4 protein was only present in the AR‐negative cells, PC3 and DU145 (Figure 5B). In LNCaP cells treated with the synthetic androgen R1881, no major changes were observed for *MCT1* and *MCT4* mRNA expression levels during up to 24 h of treatment (Figure 5C, D). While MCT1 has emerged as a new anticancer target and a first MCT1 inhibitor is currently entering clinical trials 29, 30, little is known about its regulation by typical parameters of the tumour microenvironment. Figure 5E–H shows the expression levels of MCTs in LNCaP and PC3 cell lines when grown under different oxygen conditions. The results clearly show an increase in MCTs expression with hypoxia, a typical feature of the tumour microenvironment. Figure 5K, L, shows a comparison of SLC16A1 and SLC16A3 peaks from ChIP‐seq in LNCaP cell lines growing under different oxygen conditions. While no HIF‐1α binding peaks are seen for SLC16A1 (Figure 5I), there is a clear HIF1A binding peak upstream of the SLC16A3 locus (Figure 5J). Figure 5M shows a Venn diagram demonstrating the overlap between groups of genes expressed by LNCaP cells under different conditions (M Tran, unpublished results). It is interesting to observe that *SLC16A3*, *LDHA*, *PDK1* and *CA9*, previously associated with poor prognosis, appear in the group of genes expressed when hypoxia is present, with or without androgen stimulation.

### Silencing of MCTs affects the growth kinetics of prostate cell lines and decreases the expression of genes associated with poor prognosis in PCa

**Figure 6:**
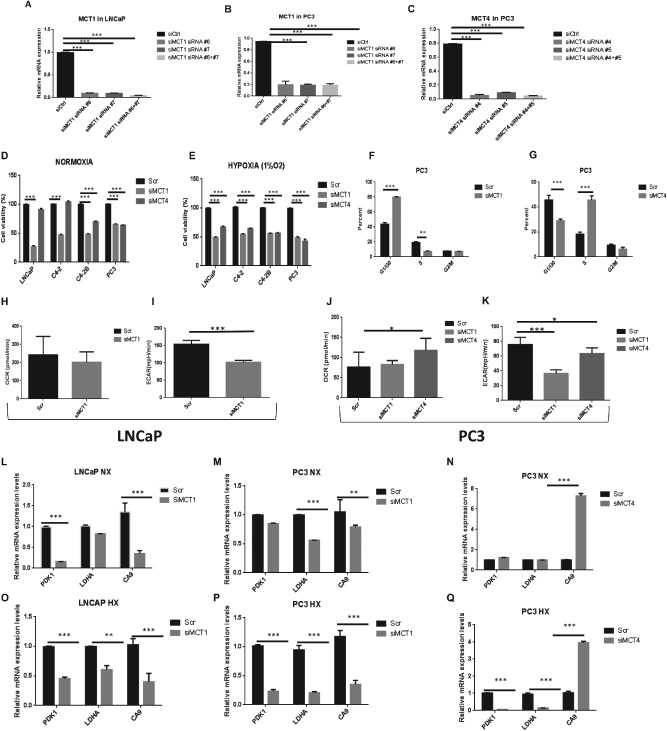
MCT knockdown affects cell viability and metabolic behaviour of PCa cell lines. (A–C) LNCaP and PC3 cells were transfected with two different combinations of siRNA directed against MCT1; PC3 cells were also transfected with two different combinations of siRNA directed against MCT4; successful knockdown was validated using qRT–PCR, 3 days after transfection (n = 4). (D–E) Effects of MCT1 (siRNA #6 + #7) and MCT4 (siRNA #4 + #5) knockdown on cell growth: (D) effect of MCT1 knockdown and MCT4 on different prostate cancer cell lines when compared to the scrambled siRNA after 48 h; (E) effect of MCT1 and MCT4 inhibition on cell growth under hypoxic conditions; ***p < 0.001. (F–G) Percentage of PC3 cells in G_0_–G_1_, S and G_2_–M fraction when MCT1 (F) or MCT4 (G) are silenced compared to scrambled (Scr); data are expressed as mean ± SD (n = 2); ***p < 0.001. (H–K) Oxygen consumption rates (OCRs) and extracellular acidification rates (ECARs) are shown for LNCaP and PC3 cell lines under control conditions (UT) and when MCT1 (siMCT1) or MCT4 (siMCT4) are silenced; *p < 0.05. (L–Q) To assess the effects of MCT1 and MCT4 knockdown on HIF‐target genes, LNCaP and PC3 cells were transfected with siRNAs and grown for 3 days: mRNA expression was assessed by qRT–PCR; values are depicted relative to vehicle control for each siRNA (n = 4); HX, hypoxia; NX, normoxia

Figure 6 shows that in different models of PCa progression, silencing of MCTs produces differential effects. Successful knockdown was validated using qRT–PCR 3 days after transfection (Figure 6A–C). We observed that, under normoxia, the silencing of *MCT1* caused a significant decrease in the viability of almost all prostate cell lines, whereas silencing of *MCT4* mostly affected the viability of the highly tumourigenic models, C4‐2B and PC3 cells (Figure 6D). Due to the already mentioned importance of hypoxia with subsequent activation of HIF‐1α and the implication in tumour metabolism, plus the observation that *MCT4* mRNA and protein levels clearly increase in hypoxic conditions, we assessed the effects of MCTs knockdown under hypoxic conditions (Figure 6E). These results demonstrate that the sensitivity of cells to *MCT4* knockdown is increased under these conditions. In addition, contrary to the results seen with normoxia, *MCT4* knockdown under hypoxia was shown to also decrease the viability of LNCaP cells (Figure 6E).

In order to define the nature of the response to silencing of *MCT1* and *MCT4*, the cell‐cycle profile of cells cultured under normoxic or hypoxic conditions were determined by flow cytometry (only data for normoxic conditions are presented, since the results were similar). A significant increase in the proportion of cells with sub‐G_1_ DNA content was detected following *MCT1* knockdown in PC3 cells (Figure 6F). After *MCT4* knockdown, the cell‐cycle profile of cells was also affected, with a clear increase in the subpopulation of cells in S phase under both growing conditions (Figure 6G). Also, in terms of metabolic phenotype, *MCT1* knockdown decreased the ECAR levels in LNCaP cells (Figure 6I), whereas *MCT4* knockdown increased OCR in PC3 cells under normoxia (Figure 6J). Also, ECAR levels decreased in PC3 cells when *MCT1* and *MCT4* were silenced, even under normoxia (Figure 6K).

Following this observation, we used qRT–PCR to compare mRNA levels of the metabolism‐related proteins, namely those previously linked to a poorer outcome, before and after *MCT1* and *MCT4* knockdown (Figure 6L–Q). We found that, under normal oxygen levels, *MCT1* knockdown decreased PDK1 and CA9 in LNCaP cells and LDHA and CA9 in PC3 cells, whereas under the same conditions, *MCT4* knockdown did not affect PDK1 and LDHA levels and actually resulted in an increase in CA9 levels. In contrast, when cells were grown under low‐oxygen conditions, *MCT1* knockdown caused a decrease in PDK1, LDHA and CA9 levels in LNCaP and PC3 cells. The same was observed when *MCT4* was silenced in PC3 cell lines. *MCT4* knockdown also caused a drastic increase in CA9 levels in PC3 cells. These results show a clearly distinct effect caused by MCTs knockdown in PCa cells under different microenvironmental conditions.

### MCT1 and MCT4 expression is high in PTEN‐negative and pRb p53‐negative tumours and the use of AR‐C155858 decreases proliferation and increases cell death in mouse tumours

**Figure 7:**
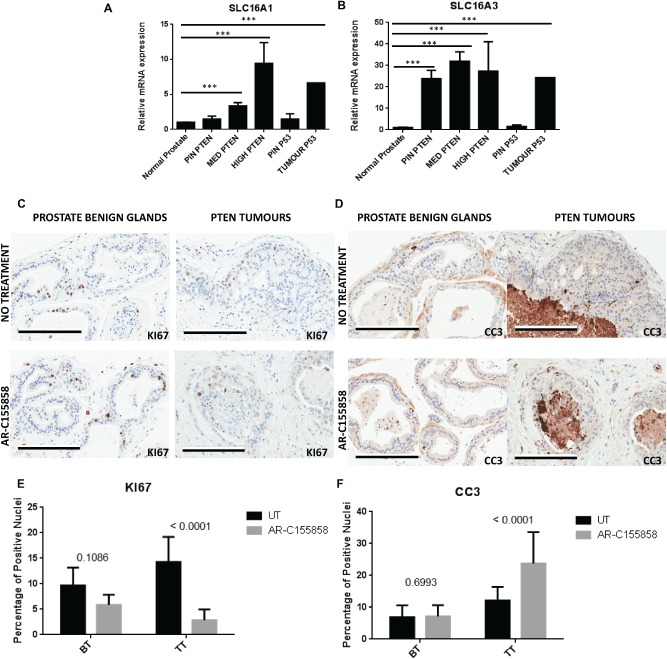
(A, B) SLC16A1 (MCT1) and SLC16A3 (MCT4) mRNA expression levels in tissue samples derived from transgenic mice; expression levels were assessed in PTEN‐ and p53pRb‐negative transgenic mice. Normal prostate, precursor lesions (PIN) derived from both PTEN‐ and p53pRb‐negative mice, micro‐invasive and well‐differentiated adenocarcinoma (MED PTEN), moderate and undifferentiated adenocarcinomas (HIGH PTEN) and undifferentiated adenocarcinoma in p53pRb‐negative (TUMOUR p53) were considered. (C, D) Immunohistochemistry showing ki67 (C) and cleaved caspase 3 staining (D) in mouse prostate. Benign glands and PTEN‐negative‐derived tumours are shown, with and without treatment with AR‐C155858; bars = 200 µm. (E, F) A total number of 30–50 randomly chosen images were used for the analysis of ki67 and CC3 staining in benign tissue (BT) and tumour tissue (TT); (E) percentage of positive nuclei for ki67 expression in benign tissue (BT) and tumour tissue (TT), with and without treatment; (F) percentage of nuclei positive for CC3 expression in benign tissue (BT) and tumour tissue (TT), with and without treatment

Using transgenic mouse models, we observed that SLC16A1 and SLC16A3 were both over‐expressed from normal mouse prostate to PIN lesions and medium and advanced *PTEN*‐null tumours (Figure7A, B). Also, tumours derived from p53 mice expressed high levels of SLC16A1 and SLC16A3. Interestingly, only PIN lesions from the *PTEN*‐null models, but not the *p53*‐null model, expressed higher levels of SLC16A3 compared to the control mice. This is particularly interesting, since it is known that only the PIN lesions derived from *PTEN* mice are able to progress to PCa, in contrast to PIN derived from *p53* (Figure 7A, B).

To achieve a more realistic insight into the efficacy of MCTs inhibition in PCa, we used AR‐C155858. The reason for selecting this compound relies on the fact that this small molecule is described as isoform‐specific for MCT1 and is now under clinical trials 29, whereas so far there are no specific inhibitors for MCT4. The binding site for AR‐C155858 is contained within the C‐terminal half of MCT1 and involves transmembrane (TM) domains 7–10 31. Tumours from *PTEN*‐negative mice were treated *ex vivo* with AR‐C155858 for 5 days, fixed and the presence of cleaved caspase‐3 (CC3) and KI67 levels were assessed by immunohistochemistry (IHC). Figure 7C, D shows expression of KI67 (C) and CC3 (D). We have clearly observed that benign glands were not affected by the drug. Proliferating cells are seen in benign glands in both the presence and absence of AR‐C155858. However, in tumours a decrease in KI67 expression was observed following treatment (Figure 7C). Regarding CC3, the benign glands were not affected (just 1% of apoptotic cells was verified) in contrast to *PTEN* tumours which, when treated with the drug, showed high expression of CC3 with an evident effect not only in the epithelial cells but also in the reactive stroma (Figure 7D). The quantification of KI‐67‐ and CC3‐positive nuclei for tumour tissue samples (TT) and benign tissue (BT), with and without treatment (untreated, UT) is shown in Figure 7E, F. This confirmed that AR‐C155858 caused a decrease in proliferation (*p <* 0.0001) and increased apoptosis (*p <* 0.0001) in tumour tissues, with no significant effect on benign tissue (*p =* 0.1086 and *p =* 0.6993, respectively).

## Discussion

A fundamental challenge in PCa research involves understanding the transition of cancer cells from androgen dependence to androgen independence. Hypoxia is thought to participate in androgen resistance under conditions of androgen deprivation via the induction of androgen hypersensitivity 32 and the amplification of androgen receptor activity 33. While numerous studies have investigated the involvement of altered cellular metabolism in various tumours, little is known about the metabolic alterations during PCa progression. Ranasinghe *et al.*(34) linked hypoxia with aggressiveness and metastasis in PCa. However, how the expression of proteins involved in cellular energetic metabolism is altered during prostate malignant transformation and progression, in addition to the significance of their expression, is largely unknown.

We consistently showed that proteins involved in the glycolytic pathway aberrantly over‐expressed across prostate malignancy, associated with both prostate metastatic tissue and poor prognosis. Accordingly, the highly metastatic models were shown to consume more glucose, with consequent lactate production, compared to the poorly metastatic models. Additionally, we found evidence for less energetically efficient respiration in the high metastatic lines that over‐express UCP2 protein, indicating a coupling defect in oxidative phosphorylation in these cells. These results support the concept that PCa does not fit the traditional model of a metabolic switch to glycolysis from non‐malignant to malignant cells, but instead this metabolic switch is more likely to be involved in advanced stages of the disease.

Following these observations, we found that MCT4 expression at both gene and protein levels was always associated with malignancy. Both isoforms were shown to increase under hypoxic conditions; however, only a direct link for HIF‐1α and MCT4 could be provided, with the existence of HIF‐1α binding sites present upstream of the *SLC16A3* gene. Consistent with the idea of a metabolic phenotype stimulated by hypoxic conditions that can allow the tumour to grow in the absence of androgens, our expression arrays showed that the proteins previously associated with poor prognosis in human tumour samples were the ones encoded by genes that were up‐regulated in low‐oxygen conditions, independent of the presence of androgens. Silencing *MCT4* was shown to be effective in PCa cell lines when under hypoxia. In contrast, *MCT1* silencing was shown to be effective when the cells were growing under both conditions.

Importantly, the metabolic phenotype of PCa cells was clearly affected after MCTs silencing, indicating a role for MCTs as metabolic modulators. One of the most interesting results observed was that MCTs inhibition was able to affect the expression levels of the glycolysis‐related proteins previously associated with poor prognosis in PCa. Surprisingly, an increase in CA9 was observed when *MCT4* was silenced. We believe this could be due to a possible interaction between CAIX and MCT4 34 that is disrupted by *MCT4* knockdown.

In accordance with observations in human samples, MCT4 levels increase in the transgenic mouse model tumours. However, MCT4 levels were already high in mouse PIN lesions; this high level was not found in human models. Considering that most PIN lesions derived from *PTEN*‐transgenic mice will progress to tumour, in contrast to PIN lesions derived from the *p53*‐transgenic models 35, the high levels of SLC16A3 only in PIN lesions from *PTEN* models corroborates once more the involvement of MCT4 in disease progression.

MCTs have been extensively described at the plasma membrane of solid tumours as major players in the acid‐resistant phenotype of tumour cells, and as attractive targets for cancer therapy, alone or in combination with other drugs targeting metabolism 36, 37, 38. However, their role in tumours that do not rely primarily on glycolysis, such as prostate cancer, has been largely neglected. The use of MCT1 inhibitor AR‐C155858 revealed the potential of this target as a specific anti‐tumour therapy affecting apoptosis and proliferation in tumours, with no adverse effects seen in benign glands. In addition to the promising results shown with *MCT1* inhibition, we hypothesize that the function of *MCT4* is also of great importance for pH regulation and growth in the context of *in vivo* metabolism when tumour cells are exposed to a hypoxic microenvironment, and therefore therapies targeting *MCT4* specifically should also be explored. To the best of our knowledge, this is the largest study demonstrating the metabolic heterogeneity of PCa using human samples, with clinical–pathological significance, and demonstrates a role for glycolysis‐associated proteins as putative prognostic biomarkers in PCa. Our study also positions *MCT4* as a putative prognostic biomarker in PCa, and both *MCT1* and *MCT4* isoforms as possible therapeutic targets for advanced tumours in patients.

## Author contributions

NPG conceived and carried out experiments and was involved in the study design, data collection, data analysis, data interpretation, literature search and generation of figures; SF, CEM, RC, SSS, SJ, MA and EO carried out experiments; JRV, ARM, ALC, DEN and LGDF were involved in data analysis and interpretation; MT and CS were involved in data collection; VM, FB, LGDF and DEN were involved in all the study design and data interpretation. All authors were involved in writing the paper and had final approval of the submitted and published versions.
